# 肺癌胰腺转移的临床特点及预后分析

**DOI:** 10.3779/j.issn.1009-3419.2017.08.02

**Published:** 2017-08-20

**Authors:** 建春 段, 蕊 万, 剑钦 沈, 叙仪 刘, 洁 王, 军 赵, 梅娜 吴, 鹭 杨, 彤同 安, 庆志 郭

**Affiliations:** 1 100021 北京，中国医学科学院肿瘤医院肿瘤内科 Department of Oncology, Cancer Hospital Chinese Academical of Medical Science, Beijing 100021, China; 2 353000 南平，南平市人民医院肿瘤内科 Department of Oncology, The People Hospital of Nanping, Nanping 353000, China; 3 100142 北京，北京大学临床肿瘤学院北京肿瘤医院 胸部肿瘤内一科 The First Department of Thoracic Oncology, Beijing University School of Oncology, Beijing Cancer Hospital, Beijing 100142, China

**Keywords:** 肺肿瘤, 胰腺转移, 胰腺炎, 综合治疗, Lung neoplasms, Pancreatic metastasis, Pancreatitis, Multimodality therapy

## Abstract

**背景与目的:**

由于缺乏特异的临床症状，70%的肺癌患者确诊时为局部晚期或晚期，多数患者伴有实性器官转移，不同部位转移患者的临床表现及预后不同。随着诊断技术的发展，肺癌伴胰腺转移似有逐渐增多趋势。本研究针对肺癌胰腺转移的临床特点、诊治、预后及生存情况进行统计分析，探讨肺癌胰腺转移的相关预后因素。

**方法:**

回顾性分析1996年7月-2017年6月于北京肿瘤医院胸部肿瘤内一科就诊的35例经病理确诊的肺癌并胰腺转移或在治疗过程中出现胰腺转移的患者，其中33例有完整随访资料。

**结果:**

35例患者中，小细胞肺癌28例（80%），腺癌3例（8.6%），鳞癌4例（11.4%）。初治时即存在胰腺转移者21例（60%），14例治疗过程中出现胰腺转移（40%）。在胰腺转移灶中，胰头转移者15例（42.9%），胰腺体尾部转移者20例（57.1%），单发转移23例（65.7%），多发转移12例（34.3%）。肺癌胰腺转移患者多无明显临床症状，本组病例中，仅4例患者在病程中出现胰腺炎症状。病理类型是影响肺癌胰腺转移患者生存的独立预后因素。

**结论:**

部分晚期肺癌患者可以出现胰腺转移，多见于小细胞肺癌。肺癌患者出现胰腺转移，治疗原则以全身化疗为主的综合治疗。病理类型是影响肺癌胰腺转移患者生存的独立预后因素。

## Abstract (en)

**Background and objective:**

Lacking of typical symptoms, more than 70% of patients with lung cancer are diagnosed as advanced-stage disease. Patients suffer from solid organs metastasis with different clinical features and prognosis. With development of new technology, more and more lung cancer patients are diagnosed with pancreatic metastasis. The aim of this study was to investigate clinicopathologic and survival difference by retrospective analysis among lung cancer patients with pancreatic metastases.

**Methods:**

Of the patients with lung cancer diagnosed by pathology and thorough staging evaluation and treated at Beijing Cancer Hospital with long follow-up during July 1996 and June 2017, 35 cases had pancreatic metastases.

**Results:**

There were 28 cases diagnosed as small cell lung cancer, 3 cases diagnosed as adenocarcinoma and 4 cases diagnosed as squamous cell carcinoma. There were 15 cases with pancreatic metastases in head of pancreas and 20 cases in body and tail of pancreas, 23 cases presented with isolated metastasis and 12 cases with multiple metastases. Pathological type was prognostic factor for lung cancer patients with pancreatic metastases.

**Conclusion:**

Pancreatic metastases represents an uncommon site of extrathoracic spread of disease for part of patients with advanced lung cancer. Lung cancer with pancreatic metastases should be treated by combined therapy, especially by systemic chemotherapy. Pathological type was prognostic factor for lung cancer patients with pancreatic metastases.

肺癌是全球发病率和死亡率最高的肿瘤^[1]^。我国覆盖6.5%的人口横断面调查提示，肺癌的发病率和死亡率位居全部恶性肿瘤之首^[2]^。肺癌起病隐匿，病情进展迅速，多数患者发现时已存在远处转移，常见转移部位包括脑、骨、肺内、肝等，而胰腺转移相对较少。近年，随着诊断技术的进步，肺癌胰腺转移在临床中似有增多趋势，但目前对其临床特点、治疗疗效及预后的报道较少。本研究采用单中心回顾性分析肺癌胰腺转移的临床特点、诊治、预后情况，探讨肺癌胰腺转移的相关预后因素。

## 1 资料与方法

### 1.1 一般资料

1996年7月-2017年6月北京肿瘤医院胸部肿瘤内一科共收治有完整随访资料的肺癌患者5, 016例，其中35例患者经病理活检及分期检查确诊为肺癌并胰腺转移，33例有完整随访资料。男性28例，女性7例；发病年龄30岁-73岁，中位年龄57岁；34例患者入院时美国东部肿瘤协作组（Eastern Cooperative Oncology Group, ECOG）体力状况评分为0级-2级，1例患者ECOG为3级。

### 1.2 肺癌的诊断与原发肿瘤情况

35例患者均经原发灶或转移灶活检病理明确诊断为肺癌，28例为小细胞肺癌（80%），腺癌3例（8.6%），鳞癌4例（11.4%）。完善分期检查后，25例为小细胞肺癌广泛期，3例为小细胞肺癌局限期（治疗过程中出现胰腺转移），7例非小细胞肺癌均为Ⅳ期。其中21例患者为发病时即存在胰腺转移，14例患者为治疗过程中出现胰腺转移。胰腺转移距原发灶确诊间隔时间为3个月-18个月，中位间隔时间为7.1个月。本组患者仅6例患者胰腺转移是唯一的血行转移部位，8例在胰腺转移同时分别伴骨转移（2例）、脑转移（5例）、肝转移（1例）。有2个以上脏器转移21例，最常合并的转移部位是远处淋巴结，多为腹腔和腹膜后淋巴结转移。

### 1.3 胰腺转移癌的诊断

胰腺病灶经病理确诊或在治疗及随访过程中直径变化者[经腹部增强计算机断层扫描（computed tomography, CT）或磁共振成像（magnetic resonance imaging, MRI）明确]诊断为胰腺转移。其中4例经病理证实为小细胞肺癌胰腺转移，其他均采用影像学诊断。胰腺转移的评效及后期随访均采用腹部增强CT或MRI检查。

### 1.4 统计学分析

统计学分析采用SPSS 20.0，单因素分析采用*Kaplan-Meier*生存分析法进行分析，并对结果进行*Log-rank*检验。多因素分析采用*Cox*风险比例模型。*P* < 0.05为差异有统计学意义。

## 2 结果

### 2.1 肺癌胰腺转移的临床特点

在胰腺转移灶中，胰头转移者15例（42.9%），而胰腺转移灶位于体尾部者20例（57.1%）。初治时即存在胰腺转移者21例（60%），治疗过程中出现胰腺转移者14例（40%）。本组病例中有4例患者合并胰腺炎，4例患者发现胰腺转移时出现腹胀、腹痛等症状，1例患者合并胆道梗阻症状。其余26例患者主要表现为呼吸系统相关症状，如憋气、呼吸困难等，无明显胰腺转移相关症状。

本组患者中4例合并胰腺炎患者中，1例患者为初诊时出现，3例患者中为疾病治疗过程中出现，伴有明显血尿淀粉酶升高，均经积极保守治疗后缓解。

### 2.2 影像学特点

本组患者中23例（65.7%）胰腺转移表现为胰腺单发结节，15例为初诊时伴胰腺转移，8例为治疗过程中出现；11例为胰头转移，12例为胰体尾部转移。12例（34.3%）患者表现为胰腺多发结节，胰头转移4例，体尾部转移8例。腹部CT平扫表现为胰腺的单发或多发的低密度结节，增强剂后表现为不规则强化或病灶周围轻度强化。

### 2.3 治疗

35例患者中共33例接受包括化疗和（或）放疗的综合治疗。小细胞肺癌患者一线以EP方案（依托泊苷+顺铂）为基础的联合化疗，部分病例联合紫杉醇或六甲密胺等药物。而非小细胞肺癌患者多行NP（长春瑞滨+顺铂）或GP（吉西他滨+顺铂）等标准一线方案治疗。其余2例患者因ECOG状况差，未行全身化疗。一线化疗周期数为1个-10个周期。其中4例患者在化疗间期接受胰腺三维适形放疗，放疗剂量为30 Gy-40 Gy。一线治疗过程中出现进展的患者中18例进行2线化疗，常用化疗方案分别有紫杉醇+卡铂、异环磷酰胺+拓扑替康等。

### 2.4 肺癌胰腺转移的预后因素分析

单因素分析结果显示患者的病理类型、胰腺转移出现的时间、胰腺转移数目是影响患者生存的独立预后因素（表 1）。患者的ECOG评分、胰腺转移部位、合并症、既往史、吸烟史、体重减轻与否、是否行胰腺病灶放疗、有无胰腺炎症状及胰腺治疗疗效与生存期的关系均无统计学意义。33例患者中，共有小细胞肺癌26例，非小细胞肺癌7例（腺癌3例，鳞癌4例）。分析显示，小细胞肺癌生存期优于非小细胞肺癌，*P*=0.001（生存曲线见图 1）。初诊时肺癌伴胰腺转移的患者生存要劣于在治疗过程中出现胰腺转移的患者（生存曲线见图 2）。肺癌伴单发胰腺转移患者的预后优于胰腺多发转移者（生存曲线见图 3）。

**1:** 肺癌胰腺转移患者临床病理特征与患者预后的单因素分析 The univariate analysis of clinicopathological features and prognosis in lung cancer patients with pancreatic metastasis

Clinicopathological features	*n*(%)	Median overall survival (mOS) (months)	*P*
Pathological type			0.001
Small cell lung cancer	26 (78.8)	10.3	
Non-small cell lung cancer	7(21.2)	5.7	
ECOG performance status			0.154
0-1	28 (84.8)	9.4	
2-3	5 (15.2)	8	
Diagnose time of pancreatic metastasis			0.015
First visit	20 (60.6)	8	
During treatment	13 (39.4)	11.05	
Location of pancreatic metastasis			0.225
The head of the pancreas	14 (42.4)	9.05	
The body or tail of the pancreas	19 (57.6)	9	
Number of pancreatic metastasis			0.051
Single	22 (66.7)	9.9	
Multiple	11 (33.1)	7	
ECOG: Eastern Cooperative Oncology Group.			

**1:**
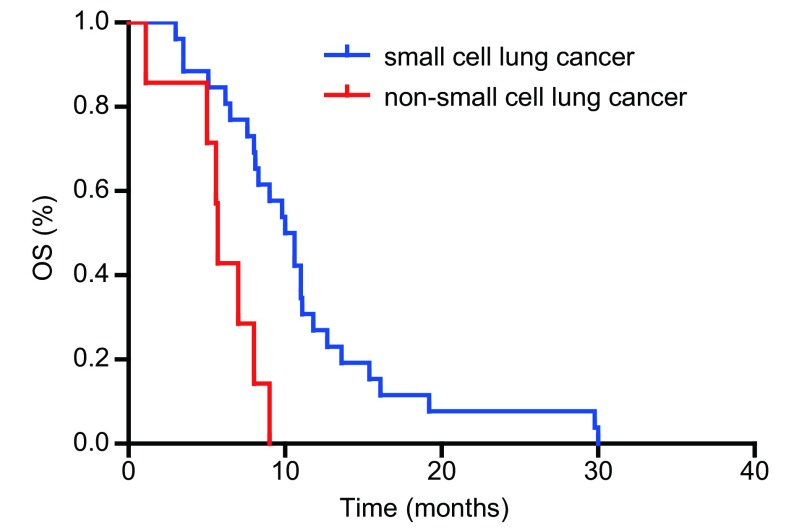
不同病理类型肺癌伴胰腺转移患者生存比较 Survival curves between lung patients with pancreatic metastasis but different pathological type

**2:**
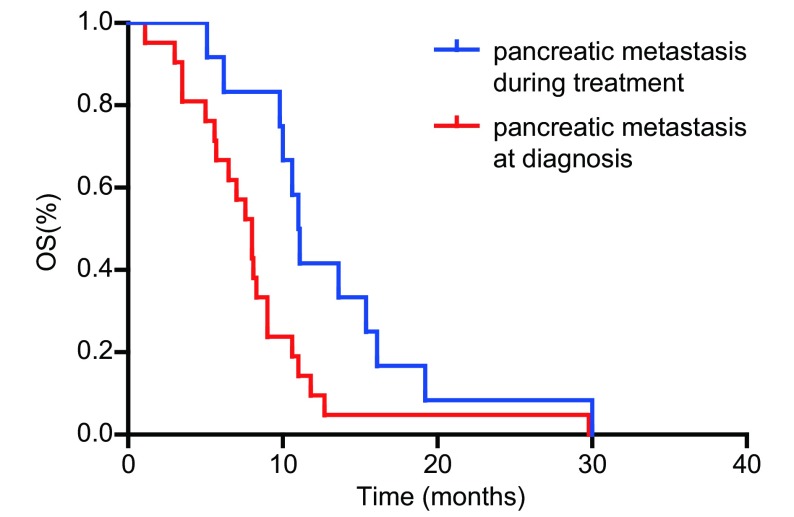
肺癌不同时间段胰腺转移患者生存比较 Survival curves between lung patients with pancreatic metastasis diagnosed at different time

**3:**
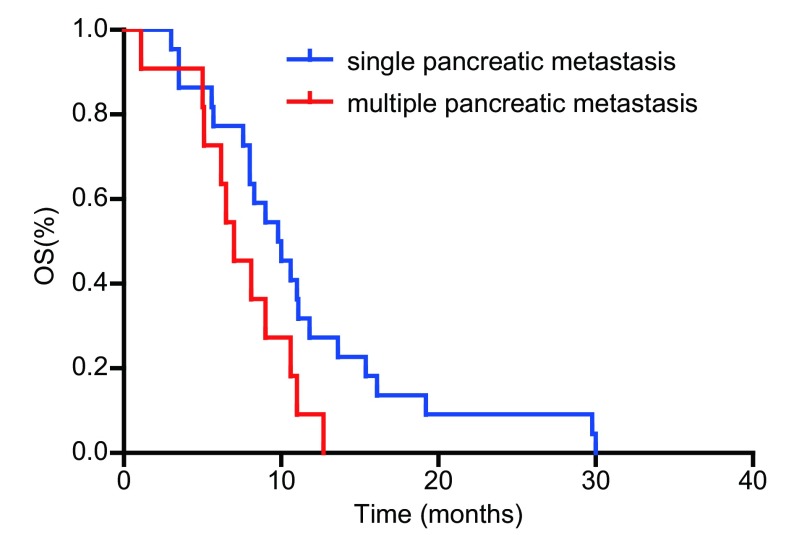
不同胰腺转移灶数目患者生存比较 Survival curves between lung patients with different number of pancreatic metastasis

将单因素分析后具有统计学意义的三项（病理类型、胰腺转移时间、胰腺转移数目）纳入多因素分析，*Cox*回归分析结果显示病理类型为影响肺癌胰腺转移患者预后的独立危险因素（表 2）。

**2:** 肺癌胰腺转移患者临床病理特征与患者预后的多因素分析 The multivariate analysis of clinicopathological features and prognosis in lung cancer patients with pancreatic metastasis

Variates	Overall survival		
	Hazard ratio	95%CI	*P*
Pathological type	0.270	0.096-0.764	0.014
Diagnose time of pancreatic metastasis	1.868	0.827-4.219	0.133
Number of pancreatic metastasis	0.467	0.212-1.028	0.059

## 3 讨论

胰腺是少见的恶性肿瘤转移部位，其高发年龄为50岁-70岁，男女比例为2:1。Z' graggen等^[3]^曾总结单中心5年内胰腺转移瘤患者的临床特点，共有10例胰腺转移瘤患者，其中肺来源者4例，出现胰腺转移的中位间隔时间为14个月-24个月。Adsay等^[4]^报道胰腺转移瘤中，最常见的原发肿瘤是肺癌，其次则为胃肠道和淋巴瘤。由此可见，与其他肿瘤相比，肺癌胰腺转移的发生几率相对较高，其中发生胰腺转移最常见的病理类型是小细胞肺癌。本组患者中28例为小细胞肺癌，占80%，可能与小细胞肺癌较易发生血行转移有关。Liu等^[5]^曾对其院1985年-2005年收治的小细胞肺癌进行回顾性分析，共14例患者发生胰腺转移，发生几率为0.63%（14/2, 212），发生胰腺转移时的中位年龄为45岁。而本组是对北京肿瘤医院1996年-2017年20年来的肺癌患者进行回顾性研究，肺癌胰腺转移的发生率为35/5, 016，约占同期肺癌患者的0.7%，与既往报道一致。

胰腺转移瘤常以原发肿瘤所引起的症状为主要表现，其转移灶的症状往往不明显。本组多数患者大部分无胰腺相关临床症状，仅有4例患者出现腹痛、黄疸等，其他症状轻微。胰腺转移瘤的相关症状包括腹痛、体重减轻和梗阻性黄疸^[6]^，上消化道出血和急性胰腺炎则较少见，而更常见于胰腺原发肿瘤。Roland和van Heerden^[7]^报道，27例有症状的胰腺转移患者中，直肠（6/27）和肺（5/27）是最常见的原发肿瘤，中位生存期短（8.7个月），生存期最长为26个月。尽管尸检报告中发现小细胞肺癌胰腺转移发生率可高达24%-40%^[8]^，但出现黄疸症状的病例在临床上罕见^[9]^，与胰腺转移相关的急性胰腺炎在临床亦不常见，Chowhan等^[10]^报道其发生率为3.3%-7.5%。本研究中，急性胰腺炎的发生率为11.4%，与既往报道相似。但对于该类患者来说，急性胰腺炎一旦发生，则为急症，故应关注此问题。胰腺转移癌可在癌症初始时以急性胰腺炎为首发症状，亦可在化疗过程中，因细胞毒药物引起胰腺转移灶坏死，释放大量相关溶解酶类，从而诱发胰腺炎。患者往往伴随血、尿淀粉酶的增高。故若疑及急性胰腺炎时，一定注意监测相应症状及实验室相关指标。何勇等^[11]^曾报道1例细支气管肺泡癌（bronchioloalveolar carcinoma, BAC）合并急性胰腺炎患者。该患者确诊BAC行手术治疗10 d后，出现急性胰腺炎症状，保守治疗略有好转，很快再次出现腹痛加重，完善腹部CT后，提示胰腺及双侧肾上腺占位，术后49 d患者因全身衰竭死亡。因此，完善全面分期检查对于肺癌患者至关重要，避免遗漏远处转移病灶而错误的行积极的局部治疗。Woo等^[12]^曾对1例出现胰腺转移的小细胞肺癌患者行个案报道。该患者因肿瘤侵及胰管而导致急性胰腺炎。作者指出，如该类患者能够耐受内镜下经胰腺导管支架置入，则可通过该种方式尽快使症状缓解，从而有利于更安全地进行下一步治疗。

肺癌胰腺转移灶的影像学表现缺乏特异性，B超多表现为胰腺的低回声结节，腹部CT既可以为单发结节，也可以表现为多发结节，多表现为没有明显强化或不规则强化^[13]^，而且肿块可以位于胰腺头部，也可以位于体部和尾部，各个部位的发病几率没有显著差别。本组病例中胰头部与胰体尾部发生比例为15/20，与原发性胰腺癌好发于胰头部不同。本组大部分患者病灶边缘清楚，而原发性胰腺癌往往边界不清，并且常常侵犯邻近血管。本研究中35例患者腹部CT提示胰腺转移，4例（11.4%）患者行活检病理证实为肺癌胰腺转移且均为小细胞肺癌。胰腺为腹膜后位器官，由于其位置毗邻重要器官和大血管，穿刺活检风险和并发症高，基于病史和影像学表现为主的诊断标准是临床更为常用的诊断标准。

肺癌患者出现胰腺转移已处于Ⅳ期，本组资料显示在发现胰腺转移癌时，大多数患者都已经有一个或多个其他部位转移，都已失去手术机会。本组通过对影响患者生存的单因素分析发现原发肿瘤的组织类型、胰腺转移时间、胰腺转移数目是影响患者预后的危险因素，多因素分析证实原发肿瘤的病理类型是影响患者预后的独立危险因素，小细胞肺癌患者明显优于非小细胞肺癌患者，中位生存时间分别是10.3个月和5.7个月（*P*=0.001）。可能与小细胞肺癌相比非小细胞肺癌肿瘤异质性低、一线化疗有效率高相关。本研究提示对于伴有胰腺转移的非小细胞肺癌患者预后较差，积极寻找有针对性的靶向治疗如针对表皮生长因子受体（epidermal growth factor receptor, *EGFR*）突变的EGFR酪氨酸激酶抑制剂（EGFR-tyrosine kinase inhibitors, EGFR-TKIs）、间变性淋巴瘤激酶（anaplastic lymphoma kinase, *ALK*）融合突变的靶向治疗是改善该类患者预后的关键因素。

本研究为回顾性分析，多数患者仅通过影像学诊断为肺癌胰腺转移，仅有11.4%的患者有明确胰腺转移的病理诊断。此外，病例数有限，对于多因素分析结果需要审慎对待。

综上所述，肺癌患者出现胰腺转移，预后较差，原发组织病理类型是影响预后的独立危险因素。对于伴有胰腺转移的非小细胞肺癌患者，积极寻找有针对性的靶向治疗可能是改善该类患者预后的关键。
